# The effectiveness of an integrated collaborative care model vs. a shifted outpatient collaborative care model on community functioning, residential stability, and health service use among homeless adults with mental illness: a quasi-experimental study

**DOI:** 10.1186/s12913-015-1014-x

**Published:** 2015-08-28

**Authors:** Vicky Stergiopoulos, Andrée Schuler, Rosane Nisenbaum, Wayne deRuiter, Tim Guimond, Donald Wasylenki, Jeffrey S. Hoch, Stephen W. Hwang, Katherine Rouleau, Carolyn Dewa

**Affiliations:** 1 Centre for Research on Inner City Health, Keenan Research Centre of the Li Ka Shing Knowledge Institute of St. Michael’s Hospital, 30 Bond St, Toronto, Ontario M5B 1W8 Canada; 2 Department of Psychiatry, University of Toronto, 250 College Street, 8th floor, Toronto, Ontario M5T 1R8 Canada; 3 Mental Health Services, St. Michael’s Hospital, 30 Bond St, Toronto, Ontario M5B 1W8 Canada; 4 Dalla Lana School of Public Health, University of Toronto, 155 College Street, 6th Floor, Toronto, Ontario M5T 3M7 Canada; 5 Centre for Research on Employment and Workplace Health (CREWH), Centre for Addiction and Mental Health, 455 Spadina Avenue, Suite 300, Toronto, Ontario M5S 2G8 Canada; 6 Centre for Excellence in Economic Analysis Research (CLEAR), Keenan Research Centre of the Li Ka Shing Knowledge Institute of St. Michael’s Hospital, 30 Bond St, Toronto, Ontario M5B 1W8 Canada; 7 Institute of Health Policy, Management and Evaluation, University of Toronto, Health Sciences Building, 155 College Street, Suite 425, Toronto, Ontario M5T 3M6 Canada; 8 Division of General Internal Medicine, Department of Medicine, University of Toronto, St. Michael’s Hospital, 30 Bond St, Toronto, Ontario M5B 1W8 Canada; 9 Global Health Program, Department of Family and Community Medicine, University of Toronto, 263 McCaul Street, 3rd Floor, Toronto, Ontario M5T 1W7 Canada; 10 Department of Family and Community Medicine, St. Michael’s Hospital, 30 Bond St, Toronto, Ontario M5B 1W8 Canada

**Keywords:** Homeless persons, Collaborative mental health care, Mental disorders, Mental health services

## Abstract

**Background:**

Although a growing number of collaborative mental health care models have been developed, targeting specific populations, few studies have utilized such interventions among homeless populations. This quasi-experimental study compared the outcomes of two shelter-based collaborative mental health care models for men experiencing homelessness and mental illness: (1) an integrated multidisciplinary collaborative care (IMCC) model and (2) a less resource intensive shifted outpatient collaborative care (SOCC) model.

**Methods:**

In total 142 participants, 70 from IMCC and 72 from SOCC were enrolled and followed for 12 months. Outcome measures included community functioning, residential stability, and health service use. Multivariate regression models were used to compare study arms with respect to change in community functioning, residential stability, and health service use outcomes over time and to identify baseline demographic, clinical or homelessness variables associated with observed changes in these domains.

**Results:**

We observed improvements in both programs over time on measures of community functioning, residential stability, hospitalizations, emergency department visits and community physician visits, with no significant differences between groups over time on these outcome measures.

**Conclusions:**

Our findings suggest that shelter-based collaborative mental health care models may be effective for individuals experiencing homelessness and mental illness. Future studies should seek to confirm these findings and examine the cost effectiveness of collaborative care models for this population.

## Background

Homelessness continues to be a major social concern throughout North America, with an estimated 150,000 individuals experiencing homelessness each year in Canada and 1.4 million in the US [1–3]. In Toronto, Canada’s largest urban center, approximately 28,000 different individuals used emergency shelters during 2008 [4], and single men outnumbered single women in shelter use, using 70 % of beds compared with 29 % for women [5].

Among individuals experiencing homelessness there is a high prevalence of mental illness and addictions. A recent systematic review reported a pooled prevalence estimate of 12.7 % for psychotic disorders and 11.4 % for mood disorders such as major depression [6]. Substance use disorders are also widespread in this population, with a pooled prevalence estimate of 37.9 % [6]. In addition, individuals facing homelessness also experience chronic medical conditions [7, 8], neurocognitive impairment [9, 10], and have higher mortality rates than people who are housed [11–13]. They face several barriers to accessing health services and appropriate disease management [11, 14, 15], and often rely on emergency department visits or inpatient hospitalizations for their health care [16–19].

Studies from the United States and Canada suggest that intensive case management (ICM), assertive community treatment (ACT) and Housing First approaches are effective interventions for people experiencing homelessness and mental illness [20–25]. The high costs associated with such specialized services have limited their availability in many urban centers, however, and the mental health needs of people facing homelessness remain largely unmet [15].

Additional, more easily available models of service delivery for this disadvantaged population are therefore urgently needed, particularly for individuals in need of less intensive models of service delivery. It has been suggested that programs that integrate both the health and social components of care are likely to be most effective [26]. Shelter-based collaborative mental health care arrangements are examples of such programs [27–29]. In collaborative mental health care models, patients, their families, their caregivers, and health providers from primary and mental health care settings work together to provide more coordinated services for individuals with mental health needs [30]. A substantial body of evidence suggests that collaborative care models are effective in reducing psychiatric symptoms and in enhancing quality of care among individuals with depression being treated in a primary care setting [31, 32]. Moreover, a growing number of studies are showing the beneficial effects of such models for other psychiatric disorders across primary care, specialty and mental health settings, including improvements in mental health and physical health symptoms, and functional outcomes [30, 33–37]. What is less well understood is the effectiveness of these models for disadvantaged populations, such as people experiencing homelessness, or key model ingredients underlying successful outcomes [35].

Although a growing number of collaborative mental health care models have been developed, few studies have utilized such interventions among homeless populations [30, 38, 37]. A study of a Shared Care Clinical Outreach Service to provide multidisciplinary care to individuals experiencing homelessness demonstrated that it is possible to adapt a collaborative mental health care model for this population [39]. A pilot study of a shelter-based collaborative mental health care team by our group showed positive housing outcomes among 49 % of participants and clinical improvement among 35 %, 6 months after program enrollment [40]. It must be noted however, that the pilot study`s sample was recruited from a single site, thus limiting the generalizability of study findings.

Although the concept of shelter-based collaborative mental health care is intuitively appealing, there is currently insufficient evidence to judge its effectiveness, or the comparative effectiveness of different models of collaborative care. This study uses a quasi-experimental design to address these knowledge gaps by comparing the outcomes of two shelter-based collaborative mental health care models [41–44] for individuals facing homelessness and mental illness: (1) an integrated multidisciplinary collaborative care (IMCC) model (liaison attachment model) and (2) a less resource intensive shifted outpatient collaborative care (SOCC) model. Studies conducted in samples of individuals with mental health conditions have shown that both types of models may increase accessibility to mental health services [36, 45, 46], improve clinical outcomes [47], reduce hospitalizations [48, 49] and result in high provider [45, 50, 51] and patient satisfaction [52, 53]. These studies, however, were not conducted in homeless populations, experiencing additional challenges in having their health and social needs met. In our study, IMCC involves a shelter-based team composed of shelter staff, psychiatrists, primary care physicians and other health professionals, whereas SOCC involves a psychiatrist providing outpatient care in a shelter setting, working closely with shelter staff, with primary care and other health supports provided off-site at community-based clinics [41–44]. The primary measure of effectiveness in this study was the patient’s level of community functioning 12 months after study enrolment. The secondary measures of effectiveness included: housing outcomes and health service utilization 12 months after study enrolment.

## Methods

### The setting

Integrated multidisciplinary collaborative care (IMCC). Agency A is a 780-bed shelter that provides care each year for over 4000 men experiencing homelessness in Toronto, Ontario. Agency A has received funding for a shelter-based multidisciplinary health team and has adopted an IMCC model of service delivery in partnership with a local teaching hospital.

In the integrated care or liaison attachment model, an on-site psychiatrist or mental health worker becomes an integral part of a primary care team [42]. In this model, shelter staff and health care providers work as a single team and share a common electronic medical record. A psychiatric consultant works onsite four half days per week at Agency A. The consultant time is divided into two-thirds direct patient care and one-third indirect patient discussion and educational support to the team members to support the provision of mental health care. The IMCC model offers increased ease of referral, an interdisciplinary stepped approach to care, increased communication between diverse providers, coordinated care plans, and more integrated and comprehensive shelter based care and case management.

Shifted outpatient collaborative care model (SOCC). Agency B is a smaller 480-bed shelter located in Toronto, Ontario, that provides assistance for men over the age of 18 with similar unmet health and social services needs. Agency B has adopted the SOCC model of service delivery [41, 44], where a psychiatric consultant, not linked administratively to the shelter, provides outpatient care in the shelter setting.

At Agency B, the consultant time, half a day per week, is divided into two-thirds direct patient care and one-third indirect patient discussion and educational support to the shelter staff. The consultant psychiatrists share an electronic medical record with select shelter staff. Primary and mental health care are not integrated, and referrals to mental health are initiated by shelter staff. Agency B does not offer primary care or nursing services on site. Instead, such services are accessed through neighboring primary care centres. Other health provider support is obtained, as needed, through referral to other community agencies. The SOCC model focuses on the provision of timely psychiatric care, and offers increased ease of referral, an increased communication between shelter staff and the on-site psychiatrist, including review of treatment progress and care coordination/case management plans.

The psychiatric consultants involved in both models were drawn from the psychiatric staff of an urban academic hospital and shared an electronic medical record. Psychiatric consultations were available within approximately two weeks of referral at each of the two shelters.

Both shelters served men with complex mental health and social needs, and received referrals from a range of organizations, including hospitals, other community organizations, probation offices, as well as self-referrals. Shelter A focused on providing on site comprehensive care, while shelter B prioritized connection to off-site, community based health services. Both programs served men on-site, transitioning to other programs and services upon housing placement. Potential study participants were referred to a research assistant by shelter staff between November 2008 and November 2010. Research personnel explained the study in detail, and obtained written informed consent. The study protocol was approved by the Research Ethics Boards at the Centre for Addiction and Mental Health and St. Michael’s Hospital.

### Study participants

Individuals were eligible for inclusion in the study if they: (1) were homeless and 18 years of age or over; (2) met criteria for one or more of the following mental disorders: schizophrenia or related psychotic disorder, bipolar affective disorder, major depressive disorder, an anxiety or substance use disorder. Participants were asked to identify their diagnoses, given by the consulting psychiatrists; (3) did not have psychiatric follow up in the community. Exclusion criteria were: (1) having a mood or psychotic disorder secondary to a general medical condition; and (2) being a danger to themselves or others.

### Data collection process

Study participants were interviewed at baseline, 6 and 12 months and received a $20 honorarium for each interview completed. Study participants were encouraged to contact research staff monthly with updated contact information and received $5 for each month they checked-in. The 12 month outcomes were considered the primary outcomes.

### Measures

Community functioning was assessed using the self-rated version of the Multnomah Community Ability Scale (MCAS). The MCAS is a standardized 17-item measure of functioning of individuals with mental illness living in the community [54, 55].

Self-reported data on residential status and residential stability (e.g., number of days spent on the streets or in shelters in the past 6 months, lifetime duration of homelessness, number of moves in the past 12 months) was collected using modified residential logs from the Community Mental Health Evaluation Initiative (CMHEI) [56, 57]. We derived a composite outcome variable that represented the total number of days in shelter or streets in the past 6 months for the residential stability model described below. Number of days in shelter or streets was assessed only at baseline as categories of < 30 days, 31–90 days and > 90 days over the past 12 months but was not used in the residential stability model because proportion of lifetime homelessness was already included.

Psychiatric symptom severity was assessed by trained interviewers using the Brief Psychiatric Rating Scale (BPRS) [58–60], a widely used outcome measure in psychiatry [61] with good inter-rater reliability [59, 62]. The BPRS consists of 24 psychiatric symptom constructs rated in a 7-point scale.

Alcohol and substance use was assessed using the Addiction Severity Index (ASI), a structured interview instrument that yields information about the severity of lifetime and (previous 30 days) drug and alcohol use [63].

Self-reported data on health care utilization, including hospitalizations in the past 6 months, emergency department (ED) visits in the past 6 months, and community physician visits in the previous 30 days were collected using a series of service use logs developed by the CMHEI.

### Statistical analysis

We estimated that sample sizes of 64 participants per group would achieve 80 % power to detect a 4.5 point difference in MCAS change from baseline to 12 months between the IMCC and SOCC groups with common standard deviation of change equal to 9.00 in both groups [64], with significance level of 0.05 [65, 66], and using a two-sided two-sample *t*-test. A half standard deviation is thought to represent a moderate effect size, in line with previous research [57, 67].

We used chi-square or Fisher’s exact tests to compare baseline characteristics of participants at the two program sites with respect to categorical variables. We examined the normality assumption of the residuals after regressing continuous variables on program site indicator, and then performed the *t*-test or the non-parametric Wilcoxon rank-sum test, as appropriate. Logistic regression was used to examine factors related to completing at least one follow-up study visit. The following covariates were included in this logistic regression model as they had been identified through univariate analyses to be associated with the outcome variable: English as the first language learned, education (those who had less than high school education), imputed baseline BPRS scores, and baseline self-reported diagnosis of schizophrenia. The following covariates were also included in the model although they were not significantly associated with the outcome variable: a baseline self-reported diagnosis of substance related disorder and at least one ED visit at baseline. To avoid deletion of important confounders from multivariate analyses [68], all the variables of interest that attained a significance level of 0.25 in univariate analyses were considered for inclusion in the final model [69, 70]. At baseline, six participants were missing 1 or 2 items on the BPRS. When calculating the BPRS score, we imputed values for missing items as the average of the available items when the total number of missing items was 2 or less, and then summed the items.

#### Regression models

Linear mixed models were used to compare programs with respect to changes in community functioning (MCAS) scores over the study period. Twenty-two participants were missing MCAS data at baseline, and all but one participant was missing 4 or less items. When calculating the MCAS score, we imputed values for missing items as the participant average of the available items when the total number of missing items was 4 or less, and then summed the items. When greater than 4 items were missing, the data were not imputed. The average of the available items for the participant has been suggested as a “reasonable choice” when the scale reliability is at least 0.70 [71]. For the current data, Chronbach’s alpha reliability was 0.70, 0.72 and 0.79 for baseline, 6 months and 12 months, respectively.

A negative binomial model with repeated measures, assuming exchangeable covariance structure was used to compare programs with respect to changes in residential stability over the study period because the variance was much larger than the mean.

We applied generalized estimating equations (GEEs) with the logit link to compare programs with respect to changes in the probabilities of hospitalizations (at least one hospitalization in the last 6 months), emergency department (ED) visits (at least one ED visit in the last 6 months), and community physician visits (at least one community physician visit in the last 30 days) over the study period. The choice of correlation structure was made using the quasi-likelihood under the independence model criterion (QIC).

For each outcome variable of interest, two regression models were calculated. In the first model we included program, time, and tested the interaction between program and time. Secondly, we added the following covariates to the multivariate model: baseline self-reported diagnosis of schizophrenia, baseline self-reported diagnosis of substance related disorder, Caucasian ethnicity, indicator of foreign-born, proportion of lifetime homeless at baseline (years homeless divided by age), imputed MCAS scores at baseline (except for the MCAS model as this was the outcome variable), and imputed BPRS score at baseline.

SAS 9.3 (SAS Institute Inc. Cary, NC, USA) and SPSS 20.0 were used for the statistical analyses and two-sided p-values < 0.05 were considered statistically significant.

## Results

### Participant characteristics

Table 1 describes the key demographic and clinical characteristics of the participant sample at baseline. In total, 142 male participants were recruited to the study: 70 from the IMCC program, and 72 from SOCC program. Only data from participants who completed the entire baseline visit were included in the analysis. The final sample included 70 participants from the IMCC model and 70 participants from the SOCC model. Participants had a mean age of 42.1 ± 10.7 years, and had been homeless for a median of 2.57 % of their lifetime. More than half of the sample was white (56 %).

**Table 1:** Baseline demographic and clinical characteristics

	IMCC (*N* = 70)	SOCC (*N* = 70)	
Characteristic	Mean (S.D) or N (%)	Mean (S.D) or N (%)	*p* Value
Demographics			
Mean age (in years)	42.3(10.8)	42.0(10.6)	0.912
Single or never married	53(75.7 %)	41(58.6 %)	0.031
Less than high school education	32(46.4 %)	23(32.9 %)	0.103
English first language learned	54(80.6 %)	53(77.8 %)	0.704
English main language	65(94.2 %)	64(91.4 %)	0.745
Foreign born	31(44.3 %)	21(30.0 %)	0.080
% Caucasian	34(49.3 %)	44(64.7 %)	0.068
Homelessness			
Median (IQR)% of lifetime homeless	4.0(15)	1.8(5)	0.001
Median (IQR) # of moves in past 12 months	1.0(1)	2.0(2)	0.097
No. nights spent on streets or in shelters in past 12 months			<0.001
≤ 30 days	12(17.1 %)	41(58.6 %)	
31-90 days	11(15.7 %)	17(24.3 %)	
> 90 days	47(67.1 %)	12(17.1 %)	
Self-reported Diagnosis			
Mood Disorder	31(44.3 %)	51(72.9 %)	0.001
Schizophrenia and Related Psychotic Disorders	41(58.6 %)	27(38.6 %)	0.018
Anxiety Disorder	15(21.4 %)	31(44.3 %)	0.004
Any Substance Use Problem	8(11.4 %)	13(18.6 %)	0.237
Addiction Severity Index			
Drug use scale	0.1(0.2)	0.1(0.2)	0.975
Alcohol use scale	0.1(0.2)	0.1(0.2)	0.445
Brief Psychiatric Rating Scale	46.2(9.9)	48.8(10.0)	0.133
Multnomah Community Ability scale	60.3(9.0)	57.3(10.1)	0.066

*IMCC* integrated multidisciplinary collaborative care, *IQR* interquartile range, *SOCC* shifted outpatient collaborative care

The prevalence of mood disorders, schizophrenia or schizoaffective disorders, and substance-related disorders in the sample was 59 %, 49 %, and 15 % respectively.

No significant group differences were observed on the MCAS, drug use and alcohol use scales of the ASI, or the BPRS at baseline. IMCC participants were more likely to be single or never married, were homeless for a greater median percentage of their lifetime, and had a higher prevalence rate of schizophrenia and related psychotic disorders. The prevalence of mood disorders and anxiety disorders was higher among SOCC participants.

### Follow-up rates

At 6 months, 58.6 % of IMCC and 71.4 % of SOCC participants were interviewed. Missing interviews at the 6-month visit resulted from participants declining (*N* = 19), not found (*N* = 21), incarcerated (*N* = 4), moved (*N* = 2), deceased (*N* = 2), or hospitalized (*N* = 1). At 12 months, 55.7 % of IMCC and 64.3 % of SOCC participants were interviewed. Missing 12-month interviews resulted from participants declining (*N* = 27), not being found (*N* = 20), incarcerated (*N* = 5), died (*N* = 2), or hospitalized (*N* = 2). There were no significant differences in the proportion of participants who completed at least one follow-up visit between the IMCC and SOCC programs (71.4 % vs. 78.6 %, *χ*2 = 0.952, df = 1, *p* = 0.33). The likelihood of completing at least one follow-up visit was higher among participants who learned English as their first language (Odds Ratio = 3.33; 95 % CI = 1.21–9.25) and lower among those who had a diagnosis of schizophrenia (Odds Ratio = 0.33, CI = 0.13–0.82).

### Primary outcome: Community functioning scores over time

**Fig. 1:**
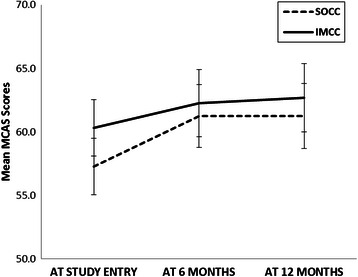
Community functioning scores over time. Mean community functioning scores obtained from the estimates in the linear mixed model with main effects of time, program and the interaction between time and program. Vertical lines correspond to 95 % confidence intervals. IMCC = integrated multidisciplinary collaborative care; MCAS = Multnomah Community Ability Scale; SOCC = shifted outpatient collaborative care

Linear mixed model analyses showed that there was a main effect of time: community functioning scores at 6 months were 3.02 units higher than at baseline (*p* = 0.0005). Moreover, community functioning scores at 12 months were 3.26 units higher than at baseline (*p* =0.0002). However, the improvement in community functioning over time did not differ between groups, as the test for the interaction between program (IMCC, SOCC) and time was not significant (Fig. 1). A self-reported diagnosis of schizophrenia was associated with greater improvement in community functioning scores over time (about 3.01 units on the average over time; *p* = 0.023). Higher BPRS values at baseline were associated with less improvement in community functioning scores over time (*p* < 0.0001); every unit of the BPRS was associated with a decrease of 0.34 units on the community functioning scale over time (Table 2).

**Table 2:** Statistical models for community functioning and no. days in shelters or streets

	MCAS Model			No. Days in Shelters or Streets Model		
Effect	β	SE	*p* Value	β	SE	*p* Value
SOCC vs. IMCC	−0.61	1.38	0.657	−0.83	0.30	0.005
6 month visit vs. Baseline	3.02	0.85	0.0005	--	--	--
12 month visit vs. Baseline	3.26	0.86	0.0002	--	--	--
6 month visit vs. 12 month visit	--	--	--	0.80	0.20	<0.0001
Schizophrenia diagnosis at Baseline	3.01	1.31	0.023	0.13	0.27	0.637
ASI Alcohol Composite Score at Baseline	−3.88	3.64	0.288	−1.11	0.72	0.123
ASI Drug Composite Score at Baseline	−7.32	8.67	0.400	−0.39	1.51	0.798
Caucasian Ethnicity	−2.27	1.43	0.114	−0.04	0.29	0.890
Foreign Born	−2.59	1.52	0.092	−0.26	0.28	0.366
BPRS at baseline	−0.34	0.06	<0.0001	−0.01	0.01	0.669
% Lifetime Homeless	−0.03	0.06	0.656	0.01	0.01	0.236
MCAS at Baseline	--	--	--	0.01	0.02	0.452

*ASI* Addiction Severity Index, *BPRS* Brief Psychiatric Rating Scale, *IMCC* integrated multidisciplinary collaborative care, *MCAS* Multnomah Community Ability Scale, *SE* standard error, *SOCC* shifted outpatient collaborative care

### Number of days in shelters or streets

**Fig. 2:**
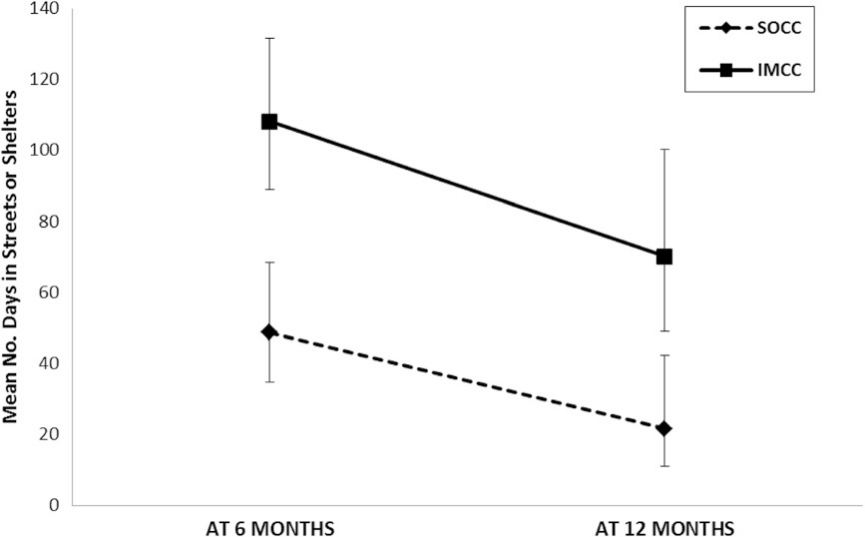
Number of days in shelters or streets over time. Mean number of days in shelters or streets obtained from estimates found in the negative binomial model with repeated measures with main effects of time, program and the interaction between time and program. Vertical lines correspond to 95 % confidence intervals. IMCC = integrated multidisciplinary collaborative care; SOCC = shifted outpatient collaborative care

A repeated measures negative binomial model showed that there was a main effect of program; the estimated mean number of days in shelters or streets was lower for SOCC participants [32.17 (95 % CI = 20.71–49.99)] relative to IMCC participants [73.88 (95 % CI = 52.96–103.04)] (Table 2). A main effect of time was also observed; the estimated mean number of days in shelters and streets improved over time: at 6 months the mean number of days in shelters or streets was 72.87(95 % CI = 58.46–90.84) and at 12 months the mean number of days in shelters or streets was 32.62 (95 % CI = 21.80–48.80). However, the improvement in number of days in shelters and streets over time did not differ between groups, as the test for the interaction between program (IMCC, SOCC) and time was not significant (Fig. 2). None of the other variables was statistically significant.

### Health services utilization

**Fig. 3:**
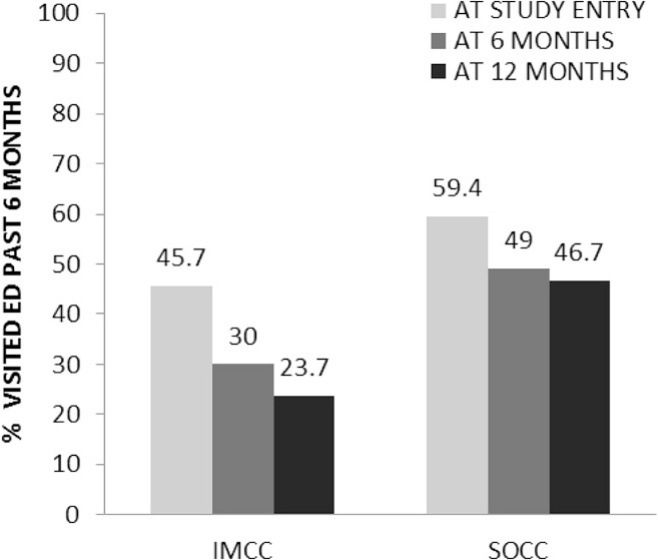
ED visits over time. IMCC = integrated multidisciplinary collaborative care; SOCC = shifted outpatient collaborative care

The proportion of patients who had at least one ED visit in the past 6 months at baseline, 6 months and 12 months is displayed in Fig. 3. The GEE analysis showed no significant interaction between program and time. However, the odds of any ED visits among SOCC participants were 1.8 times higher than for IMCC participants (OR = 1.79, 95 % CI = 1.04–3.07). Compared to baseline, the odds of an ED visit was lower at both the 6-month study visit (OR = 0.51, 95 % CI = 0.30–0.87,) and 12-month visit (OR = 0.48, 95 % CI = 0.26–0.90). The odds of any ED visit in the last 6 months was lower if the participant reported they received a diagnosis of schizophrenia (OR =0.50, 95 % CI = 0.30–0.84) (Table 3).

**Table 3:** Health service use generalized estimating equation models

	Any ED Visits in the Past 6 months			Any Overnight Hospital Visits in the Past 6 months			Any Community Physician Visits in the Past 30 days		
Effect	Odds Ratio [Exp(β)]	95 % CI	*p* Value	Odds Ratio [Exp(β)]	95 % CI	*p* Value	Odds Ratio [Exp(β)]	95 % CI	*p* Value
SOCC vs. IMCC	1.79	1.04–3.07	0.035	3.15	1.61–6.17	0.001	2.54	1.39–4.63	0.002
6 M vs. Baseline	0.51	0.30–0.87	0.014	0.45	0.26–0.79	0.006	1.31	0.73–2.35	0.358
12 M vs. Baseline	0.48	0.26–0.90	0.021	0.33	0.17–0.63	0.001	2.07	1.14–3.74	0.017
Schizophrenia diagnosis at Baseline	0.50	0.30–0.84	0.009	1.06	0.57–1.97	0.859	0.71	0.42–1.20	0.204
ASI Alcohol Composite Score at Baseline	1.01	0.19–5.43	0.992	0.55	0.15–2.05	0.369	1.23	0.29–5.20	0.777
ASI Drug Composite Score at Baseline	0.85	0.03–22.61	0.923	1.66	0.07–39.65	0.756	15.28	0.45–516.21	0.129
Caucasian Ethnicity	1.48	0.85–2.58	0.171	0.98	0.49–1.94	0.947	1.18	0.65–2.13	0.591
Foreign Born	0.86	0.49–1.53	0.611	0.86	0.40–1.86	0.697	1.20	0.65–2.21	0.555
BPRS at Baseline	1.03	1.00–1.05	0.061	1.02	1.00–1.05	0.093	0.99	0.96–1.02	0.425
% Lifetime Homeless	1.01	0.98–1.04	0.603	1.01	0.97–1.05	0.737	0.99	0.96–1.02	0.345
MCAS at Baseline	1.03	1.00–1.06	0.103	1.01	0.97–1.05	0.604	0.98	0.95–1.01	0.168

*ASI* Addiction Severity Index, *BPRS* Brief Psychiatric Rating Scale, *ED* emergency department; *IMCC* integrated multidisciplinary collaborative care, *MCAS* Multnomah Community Ability Scale, *SOCC* shifted outpatient collaborative care

**Fig. 4:**
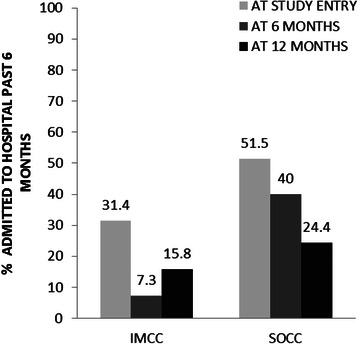
Admissions over 12 months. IMCC = integrated multidisciplinary collaborative care; SOCC = shifted outpatient collaborative care

The proportion of patients who had at least one hospitalization in the past 6 months at baseline, 6 months and 12 months is displayed in Fig. 4. The GEE models showed significant main effects of program and time, but no interaction effect. Specifically, the odds of any overnight hospital stay in the prior 6 months among SOCC participants were 3.2 times greater than for IMCC participants (OR = 3.15, 95 % CI = 1.61–6.17). The odds of an overnight hospital visit in the previous 6 months relative to baseline decreased over time; at both the 6-month study visit (Odds Ratio = 0.45, 95 % CI = 0.26–0.79) and 12-month visit (Odds Ratio = 0.33, 95 % CI = 0.17–0.63) (Table 3).

**Fig. 5:**
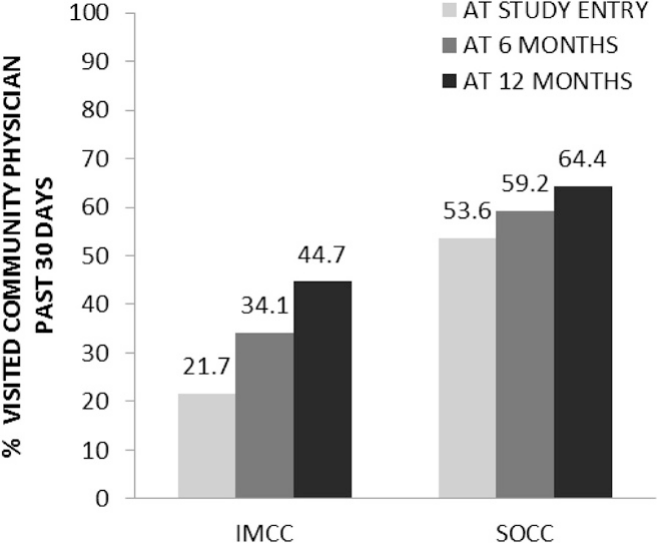
Community physician visits over time. IMCC = integrated multidisciplinary collaborative care; SOCC = shifted outpatient collaborative care

The proportion of patients who had at least one community physician visit in the past 30 days at baseline, 6 months and 12 months is displayed in Fig. 5. The GEE analysis revealed significant main effects of program, and time, however, the interaction between program and time was not significant with respect to any community physician visit in the past 30 days. The odds of any community physician visits in the past 30 days were 2.5 times greater among SOCC participants than for IMCC participants (OR = 2.54, 95 % CI = 1.39–4.63). The GEE analysis also showed significant improvements in outpatient service use over time; the odds of a community physician visit was higher at the 12-month visit relative to baseline (OR = 2.07, 95 % CI = 1.14–3.74) (Table 3).

## Discussion

The primary goal of this study was to compare the outcomes of two shelter-based collaborative mental health care models for homeless men with mental illness. We observed improvements in the entire sample over time on measures of community functioning, housing stability, and health care utilization, suggesting that the provision of shelter-based models of care may improve outcomes in this population. There was no evidence that receiving less resource intensive SOCC compared to IMCC services was associated with significantly different outcomes over the study period in terms of residential stability or health service use.

Significant improvements in community functioning, as assessed by the MCAS, were observed in both IMCC and SOCC participant groups over the follow-up period. This finding is in line with research from a previous study that compared homeless and vulnerably housed individuals with mental illness who received intensive case management or standard community services [57]. This study similarly found significant improvements in overall MCAS scores in both study groups [57]. Our findings are broadly consistent with those of a randomized controlled trial that compared an IMCC (attached liaison) model to usual care [36], conducted in a sample of participants with heterogeneous mental health conditions. The IMCC model in that study consisted of access to a team of mental health professionals (community psychiatric nurses and psychologists) who had regular contact with the general practitioner, provided patient consultations, assessments, short-term psychological treatment, and assisted with referrals for patients who needed urgent care, in addition to a team of psychiatrists who met with the mental health professionals once a month and who met regularly with the general practitioners. Usual care involved referral of patients by the general practitioner to mental health services if needed. The authors found that quality of life improved over time, and there were no significant differences between the IMCC model and usual care [36].

With regards to housing, significant reductions in the mean number of days spent in shelters and streets were observed in both groups over the follow-up period. This finding is consistent with an earlier study by Stergiopoulos et al.[40] that found that a substantial proportion of shelter residents at the IMCC site obtained housing within 6 months after referral to the program. Few studies have examined the effects of collaborative mental health interventions on housing outcomes outside of the case management and assertive community treatment literature.

Both the IMCC and SOCC programs appeared to reduce the likelihood of urgent and acute care visits and increase the likelihood of outpatient treatment engagement in the community after 12 months. These findings are consistent with prior work in this area. Bradford et al. observed significant improvements in the proportion of individuals who attended an initial follow-up appointment at a local community mental health centre following the introduction of a shelter-based intervention that provided intensive outreach of a personal support worker and weekly psychiatrist visits [27]. Podymow et al. observed reductions in the total number of ED visits among individuals facing homelessness and alcohol dependence who were enrolled in a shelter-based managed alcohol program [29]. Similarly, O’Toole et al. observed improvements in primary care use among veterans experiencing homelessness in a medical home clinical model [72]. We identified several factors associated with improvement in the study sample. Our finding that a self-reported diagnosis of a psychotic disorder at baseline was associated with improved outcomes on the MCAS is not surprising given that the MCAS was developed to assess impairments in community functioning among individuals with serious mental illness, and may better capture changes in community functioning over time in this group [54]. The likelihood of an ED visit was lower for participants with a self-reported diagnosis of schizophrenia. Although recent research has begun to examine predictors of frequent ED use [73–76], the association between certain psychiatric diagnoses (e.g., schizophrenia) and ED use among homeless populations remains to be determined. In this study, higher levels of psychiatric symptom severity at baseline were associated with poorer outcomes in community functioning over time. This finding is consistent with previous investigations that have shown an association between psychiatric symptom severity and deficits in community functioning among individuals with severe mental illness [77, 78].

In our study we did not observe any statistically significant differences in community functioning, residential stability, or health care utilization between the two models of care over time. Several factors may explain our findings. First, it is possible that the two models of care examined in this study are equally efficacious. Second, we had lower follow-up rates than expected, which may have limited the power to detect a difference over time between the two groups for each of the outcome measures. We have since implemented several approaches to improve follow-up rates with this population, including administering interviews in jails or hospitals with participants who are incarcerated or hospitalized, tracking participants through administrative databases and contact lists, and increased honoraria for monthly check ins and interviews, with excellent outcomes. Third, given the quasi-experimental design of the current study, the improvements observed over time in both IMCC and SOCC programs may reflect regression to the mean rather than true program effects. Future studies that incorporate random allocation of participants to comparison groups can help reduce the effects of regression to the mean [79]. Nonetheless, our findings suggest that a less resource intensive model may be equally efficacious 12 months following program enrolment. Key ingredients underlying successful outcomes of collaborative mental health care models are not well understood, suggesting that a better understanding of mechanisms underlying model success is essential to guide further development and replication in diverse settings [35, 37]. Although a number of key components of effective collaborative mental health care have been identified in the literature, including organizational commitment, linkages across service levels, consistently delivered interventions, staff training and supervision, care coordination and clinical monitoring with agreed upon treatment protocols, it is not clear how many, or which of those components are necessary for program success [35, 37]. The SOCC model described in this study began with a shared commitment of shelter staff and the onsite psychiatrist to improve mental health care and outcomes for shelter residents with mental health conditions. Consistent with core ingredients of effective models identified in the literature [35], SOCC improved access to psychiatric care at the shelter setting, improved communication between shelter staff and mental health specialists, and offered an opportunity for regular review of treatment progress and clinical supervision of shelter based case management plans. The model lacked the comprehensiveness and continuity of primary care embedded models such as IMCC, but was less resource intensive and easier to implement.

This study has several limitations. First, self-report measures of community functioning and health service utilization were administered in the present study. There are limitations to the validity of self-reported survey findings, and future studies that utilize clinician-rated measures of community functioning, as well as administrative databases to obtain health service use data are needed. Second, we did not collect any data related to the number of contacts with IMCC or SOCC clinicians, and it is possible that participants received variable “doses” of the intervention.

Housing First and case management programs such as ICM and ACT have been shown to improve housing stability, mental health, and substance use outcomes in this population [20, 21, 23], however, the availability of these programs is limited in many jurisdictions [15, 80, 81]. Shelter-based collaborative care models avoid long waiting lists for primary and specialty services [40], and allow for timely access to coordinated, less intensive supports. These models may have an important role in the continuum of services available to people experiencing homelessness and mental illness. As models providing comprehensive health care for participants on-site may foster dependency of the shelter system, less intensive models focused on connecting to housing and community-based treatment and rehabilitation programs may be preferable.

## Conclusions

Our findings suggest that homeless men with mental illness experience significant improvements in community functioning, housing, hospitalizations, ED visits, and community based physician visits in both shelter-based collaborative mental health care programs over time. The lack of observable differences between the programs over the study period on key outcomes suggests that less resource intensive shifted outpatient collaborative care models may offer an effective approach in many jurisdictions where the needs of homeless people with mental illness go largely unmet.
